# Residual respiratory impairment after COVID-19 pneumonia

**DOI:** 10.1186/s12890-021-01594-4

**Published:** 2021-07-17

**Authors:** Francesco Lombardi, Angelo Calabrese, Bruno Iovene, Chiara Pierandrei, Marialessia Lerede, Francesco Varone, Luca Richeldi, Giacomo Sgalla, the Gemelli Against COVID-19 Post-Acute Care Study Group, Francesco Landi, Elisa Gremese, Roberto Bernabei, Massimo Fantoni, Antonio Gasbarrini, Carlo Romano Settanni, Francesca Benvenuto, Giulia Bramato, Angelo Carfì, Francesca Ciciarello, Maria Rita Lo Monaco, Anna Maria Martone, Emanuele Marzetti, Carmen Napolitano, Francesco Pagano, Sara Rocchi, Elisabetta Rota, Andrea Salerno, Matteo Tosato, Marcello Tritto, Riccardo Calvani, Lucio Catalano, Anna Picca, Giulia Savera, Enrica Tamburrini, Alberto Borghetti, Simona Di Gianbenedetto, Rita Murri, Antonella Cingolani, Giulio Ventura, Eleonora Taddei, Davide Moschese, Arturo Ciccullo, Leonardo Stella, Giovanni Addolorato, Francesco Franceschi, Gertrude Mingrone, Maria Assunta Zocco, Mauirizio Sanguinetti, Paola Cattani, Simona Marchetti, Alessandro Bizzarro, Alessandra Lauria, Stanislao Rizzo, Maria Cristina Savastano, Gloria Gambini, Maria Grazia Cozzupoli, Carola Culiersi, Giulio Cesare Passali, Gaetano Paludetti, Jacopo Galli, Fabrizio Crudo, Giovanni Di Cintio, Ylenia Longobardi, Laura Tricarico, Mariaconsiglia Santantonio, Danilo Buonsenso, Piero Valentini, Davide Pata, Davide Sinatti, Cristina. De Rose, Luca Richeldi, Francesco Lombardi, Aangelo Calabrese, Gabriele Sani, Delfina Janiri, Giulia Giuseppin, Marzia Molinaro, Marco Modica, Luigi Natale, Anna Rita Larici, Riccardo Marano, Annamaria Paglionico, Luca Petricca, Laura Gigante, Gerlando Natalello, Anna Laura. Fedele, Marco Maria Lizzio, Angelo Santoliquido, Luca Santoro, Antonio Nesci, Valentina Popolla

**Affiliations:** 1 Division of Pulmonary Medicine, Fondazione Policlinico Universitario Agostino Gemelli IRCCS, Università Cattolica del Sacro Cuore, Largo Agostino Gemelli 8, 00168 Rome, Italy; 2 Università Cattolica del Sacro Cuore, Rome, Italy

**Keywords:** COVID, dyspnoea, cough, ABG, PFT, 6MWT

## Abstract

**Introduction:**

The novel coronavirus SARS-Cov-2 can infect the respiratory tract causing a spectrum of disease varying from mild to fatal pneumonia, and known as COVID-19. Ongoing clinical research is assessing the potential for long-term respiratory sequelae in these patients. We assessed the respiratory function in a cohort of patients after recovering from SARS-Cov-2 infection, stratified according to PaO_2_/FiO_2_ (p/F) values.

**Method:**

Approximately one month after hospital discharge, 86 COVID-19 patients underwent physical examination, arterial blood gas (ABG) analysis, pulmonary function tests (PFTs), and six-minute walk test (6MWT). Patients were also asked to quantify the severity of dyspnoea and cough before, during, and after hospitalization using a visual analogic scale (VAS). Seventy-six subjects with ABG during hospitalization were stratified in three groups according to their worst p/F values: above 300 (n = 38), between 200 and 300 (n = 30) and below 200 (n = 20).

**Results:**

On PFTs, lung volumes were overall preserved yet, mean percent predicted residual volume was slightly reduced (74.8 ± 18.1%). Percent predicted diffusing capacity for carbon monoxide (DLCO) was also mildly reduced (77.2 ± 16.5%). Patients reported residual breathlessness at the time of the visit (VAS 19.8, p < 0.001). Patients with p/F below 200 during hospitalization had lower percent predicted forced vital capacity (p = 0.005), lower percent predicted total lung capacity (p = 0.012), lower DLCO (p < 0.001) and shorter 6MWT distance (p = 0.004) than patients with higher p/F.

**Conclusion:**

Approximately one month after hospital discharge, patients with COVID-19 can have residual respiratory impairment, including lower exercise tolerance. The extent of this impairment seems to correlate with the severity of respiratory failure during hospitalization.

## Introduction

In December 2019, a novel coronavirus (SARS-CoV-2) able to infect the respiratory tract in humans emerged in Wuhan (China), causing a disease known as COVID-19. A possible complication of SARS-CoV-2 infection is a severe acute respiratory syndrome (SARS) due to interstitial pneumonia [1]. On March 11, 2020, the WHO declared COVID-19 a global pandemic. As of June, 2021 more than 175 million people have been infected by SARS-CoV-2 worldwide and 3.8 have died [2].

Several studies reported a range of clinical and laboratory features among hospitalized COVID-19 patients, including increased levels of inflammatory markers [3]. The frequency of respiratory and functional impairment after COVID-19 is still debated but several studies found reduced lung volumes, reduced diffusing capacity of the lung for carbon monoxide (DLCO) and reduced exercise tolerance following hospital discharge [4–7]. A comprehensive follow-up strategy for COVID-19 patients after clinical recovery has been advocated [8]. We performed a study to investigate the prevalence of respiratory impairment in a cohort of COVID-19 patients after hospital discharge and to determine the relationship between the severity of pulmonary involvement during hospitalization and the extent of residual clinical and functional abnormalities.

## Material and methods

### Study population and subgroups

In the post-COVID-19 outpatient program at Fondazione Policlinico Universitario “A. Gemelli” IRCCS, Rome (Italy), a multidisciplinary team evaluates patients after hospital discharge. Patients presenting between April 22nd and May 27th, 2020 were invited to participate in the study. Inclusion criteria were: previous hospitalization for COVID-19; radiological evidence of interstitial pneumonia at the time of hospital admission; nasopharyngeal swab negative for SARS-Cov-2 in the 48–72 h before study enrolment.

Based on arterial blood gas (ABG) analysis values during hospitalizzation, three subgroups were defined using the worst PaO_2_/FiO_2_ value (p/F): mild (p/F ≥ 300), moderate (≤ 200 p/F < 300), and severe (p/F < 200). Such values, derived from the Berlin Criteria for ARDS, have been used in clinical practice to stratify severity of respiratory failure [9]. Written informed consent was obtained from each participant. The study protocol was approved by the Institutional Review Board of the Università Cattolica del Sacro Cuore (Rome, Italy) (approval number 0024185/20). Clinical evaluations, exams, and procedures were performed in accordance with the Declaration of Helsinki.

### Study design and assessments

In this cross-sectional study, all patients underwent physical examination, resting ABG, pulmonary function tests (PFT) with DLCO, and 6MWT at the time of the study visit. ABG was analyzed using the ABL90FLEX radiometer (A. de Mori Spa Milano, Italy). The Biomedin Spirometer (software Baires version 5.1 revision 3, Biomedin SRL, Padova, Italy) was used to perform PFT and DL_CO_ with the single breath-hold method (software Baires version 5.1 revision 3, Biomedin SRL, Padova, Italy). Lung function tests were performed according to current international guidelines [10, 11]. The 6MWT was used to assess the sub-maximal level of functional capacity. After 6 min of rest, the patient was instructed to walk along a 50 m corridor as fast as possible for 6 min wearing a finger/forehead pulse oximeter (Nonin 3100 Wristox pulse oximeter with nVISION software; Nonin Medical Inc, Plymouth, MN, USA) to record percutaneous oxygen saturation (SpO_2_) and heart rate (HR). At the end of the 6 min (or before, if the patient was unable to walk any further for fatigue, dyspnoea, or chest pain, or if saturation dropped below 80%) the distance covered was recorded and the patient was invited to sit and rest for 6 min. A drop in oxygen saturation ≥ 4% from baseline was considered to be clinically relevant During the study visit, a visual analog scale (VAS) score was used to measure the levels of dyspnoea and cough. Using a 100 mm linear scale, where 0 mm represents absence and 100 mm represents the worst dyspnoea and cough, patients were asked to report the levels of these two symptoms at the onset of the disease (i.e. immediately before hospital admission), during hospitalization, and at the moment of the study visit [12]. Retrospective data collected for this study included chest imaging findings, pharmacological treatments, p/F values, the type of respiratory support, and duration of hospitalization.

### Statistical analysis

Descriptive statistics such as means with standard deviations or medians with interquartile ranges were used for continuous variables after checking for normal distribution of data. Frequencies or percentages were used to describe categorical variables. Dyspnea and cough VAS scores collected from the same patient were compared using analysis of variance (ANOVA) for repeated measures. Between-group comparisons of demographics and clinical data were performed using one-way analysis of covariance (ANCOVA) for continuous variables and the chi-square test for categorical variables. Estimated means of physiological variables across study groups were reported after adjustment by age, included as covariate in the ANCOVA model due to a significant age difference between study groups. P-values less than 0.05 were considered statistically significant. SPSS (version 24, IBM, New York, NY, USA) was used to perform statistical analyses.

## Results

### Characteristics of the study population

One hundred and fifty-seven patients included in the post-Covid follow-up program were screened for inclusion in the study. Twenty-six patients were excluded due to positivity at the nasopharyngeal SARS-Cov-2 swab; eighteen patients were excluded due to lack of radiological evidence of COVID-19 pneumonia at the time of hospitalization; twenty-seven patients were excluded because they had not been hospitalized (discharged from the Emergency Department). Eighty-six patients were included in the analyses.

The characteristics of the study population are reported in Table 1. Study visit occurred 35 (SD: ± 21) days after hospital discharge. At the time of hospital admission, chest imaging (i.e., chest X-ray or chest CT scan) revealed bilateral ground-glass opacities (GGO) with or without consolidations in 70 patients (81%). Sixteen patients (19%) had unilateral lung involvement. During hospitalization, 56 (65%) patients required supplemental oxygen and 15 patients (17%) were admitted to the intensive care unit.

**Table 1:** Characteristics of patients during the hospitalization for Covid-19

	Available observations	N = 87
Age, years		58 (13)
Male, n (%)		58 (67)
BMI (kg/m^2^)		26.7 (4.4)
p/F worst	76	281 (150)*
Hospitalization time (days)	86	13 (10)*
Day from discharge (days)	85	35 (21)*
Smoking history, n (%)	85	
Never smoker		33 (39)
Smoker		4 (5)
Former smoker		48 (56)
Pulmonary disease history, n (%)	86	
COPD		3 (4)
Asthma		9 (11)
Radiology (chest XR or CT), n (%)	86	
Chest CT performed		51 (59)
Unilateral involvement		16 (19)
Bilateral involvement		70 (81)
Antiviral therapy, n (%)	86	
Lopinavir/Ritonavir		37 (43)
Darunavir/Ritonavir		53 (62)
Anti-IL-6, n (%)	86	31 (36)
Enoxaparin, n (%)	86	42 (49)
Azithromycin, n (%)	86	41 (48)
Hydroxychloroquine, n (%)	86	81 (94)
Corticosteroids, n (%)	86	6 (7)
Respiratory support, n (%)	86	
Ventimask		56 (65)
HFNC		9 (11)
NIV or CPAP		13 (15)
Orotracheal Intubation		6 (7)
ICU admission, n (%)	86	15 (17)
FVC	83	
Litres		3.9 (1.1)
% predicted		104.6 (18.5)
FEV-1	83	
Litres		3.1 (0.9)
% predicted		102.8 (16.0)
FEV-1/FVC	83	
% predicted		79.6 (5.8)
TLC	82	
Litres		5.7 (1.3)
% predicted		89.6 (14.6)
DLco	83	
Litres		21.2 (6.8)
% predicted		77.2 (16.5)
RV	82	
Litres		1.58 (0.47)*
% predicted		74.8 (18.1)
RC/TLC	82	
Ratio		30 (10)*
% predicted		79.0 (13.0)
ABG	84	
pO2 (mmHg)		91.4 (8.0)
pCO2 (mmHg)		38.8 (3.1)
pH		7.42 (0.02)*
d(A-a) (mmHg)		13.0 (7.5)
6MWT	82	
SpO2 basal		97 (2)*
SpO2 nadir		95 (4)*
Meters		500 (88)*

Categorical data are presented as counts (%). Continuous data are presented as means with standard deviation (SD) or medians with interquartile range (IQR) for non-normally distributed variables (indicated with *). BMI: Body Mass Index; ICU: Intensive Care Unit; COPD: Chronic Obstructive Pulmonary Disease; IL-6: Interleukin; HFNC: High Flow Nasal Cannula; NIV: Non-Invasive Ventilation; CPAP: Continue Positive Airway Pressure

**Fig. 1:**
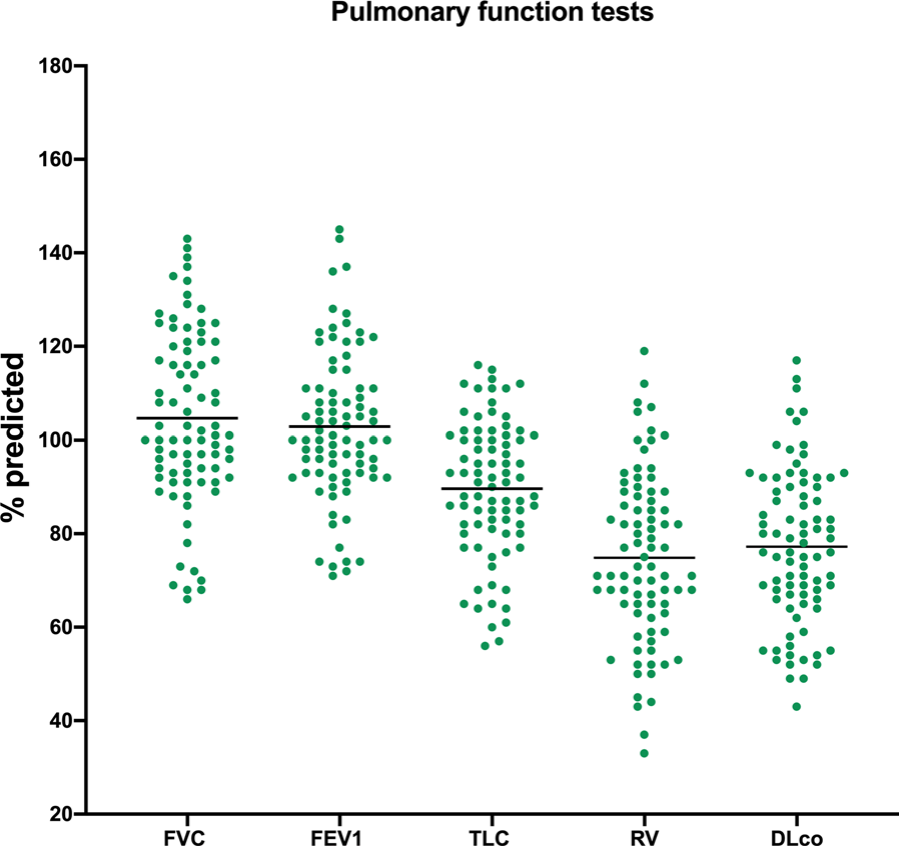
Overall cohort pulmonay function tests. Total Lung Capacity (TLC), Forced Vital Capacity (FVC), Forced Expiratory Volume in the first second (FEV-1), Residual Volume (RV) Diffusion Lung capacity for carbon monoxide (DLco)

Pulmonary function testing (Fig. 1) showed overall preserved lung volumes, with mean percent predicted total lung capacity (TLC) of 89.6% (± 14.6%) and mean percent predicted forced vital capacity (FVC) of 104.6% (± 18.5%). Mean percent predicted forced expiratory volume in one second (FEV_1_) was 102.8% (± 16.0%). Mean percent predicted residual volume (RV) was the only respiratory volume reduced under the 5th percentile (74.8 ± 18.1%). Percent predicted DLco was also mildly reduced (77.2 ± 16.5%).

**Fig. 2:**
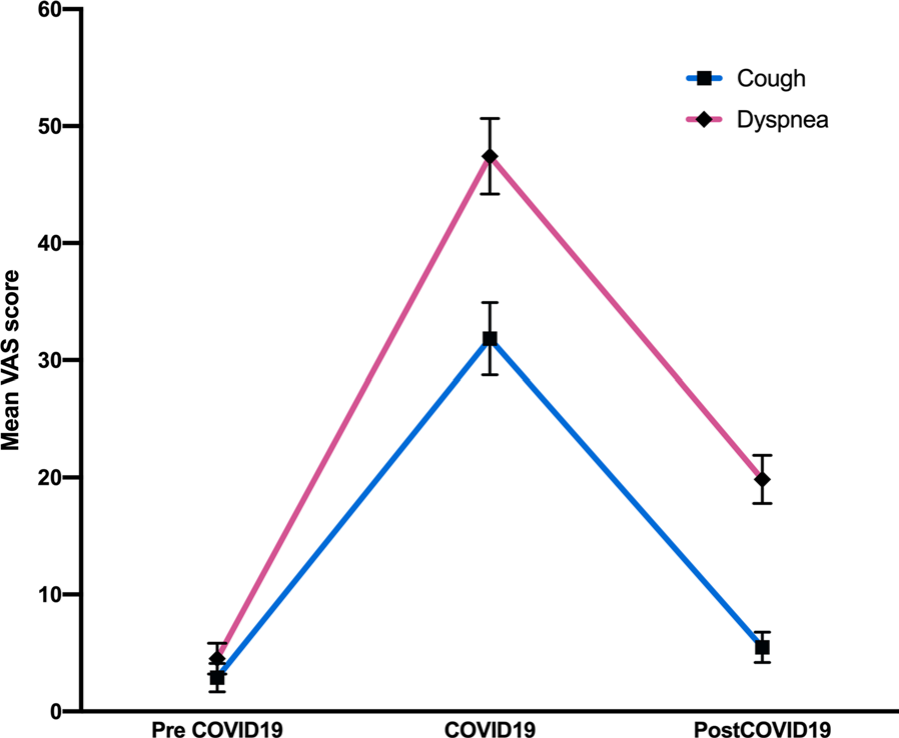
Overall cohort trends of the VAS scores for dyspnoea and cough before hospitalization, during hospitalization, and at follow-up

Mean partial pressure of oxygen (pO_2_) was 91.4 mmHg (± 8.0) and mean alveolar-arterial oxygen gradient (d(A-a)) was 13.0 mmHg (± 7.5). Approximately one month after hospital discharge, patients reported more dyspnoea than pre-admission values (VAS score estimated mean difference: 15.3 mm; p < 0.001) (Fig. 2; Table 2).

**Table 2:** Dyspnoea and cough in overall population in three-time points: before hospitalization, during the hospitalization, and at study visit-time; VAS: Visual Analogic Scale

	Before hospitalization	During Covid-19 hospitalization	Visit-time	*p* value
Dyspnoea VAS	4.5 (1.3)	47.4 (3.2) °^#^	19.8 (2.1) ^#^	< 0.001
Cough VAS	2.9 (1.2)	31.8 (3.1) °^#^	5.5 (1.3)	< 0.001

Data are reported as estimated means (Standard Error) after adjustment for age used as covariate in the ANOVA model. ° p value < 0.05 *vs* “before hospitalization”. ^#^ p value < 0.05 *vs* “visit-time”

### Comparison of study groups by p/F ratio

Most patients (n = 76, 88%) had an ABG performed during the hospitalization and were therefore included in this analysis. Among excluded patients, 8 patients had no ABG and 2 patients had an ABG performed with unknown oxygen inhaled fraction.

Gender, smoking status and comorbidities were not different across groups. Six patients (21%) in the mild hypoxemia group had a history of asthma. Patients in the severe hypoxemia group were older (63.1 years, p = 0.014 vs other groups), had a longer hospitalization time (23.0 days, p < 0.001 *vs* other groups) and were treated with anti-IL-6 drugs and enoxaparin more frequently (respectively 81% and 95%, p < 0.001 *vs* other groups).

Lung volumes were generally lower in the severe hypoxemia group, including lower percent of predicted FVC (p = 0.005), lower percent of predicted FEV1 (p = 0.009) and lower percent of predicted TLC (p = 0.012) (Table 3). In the severe hypoxemia group mean percent predicted TLC was 80.4% (± 3.1), indicating a residual restrictive impairment after 35 days from hospital discharge. DLco was also more reduced (64.9 ± 3.2% predicted) in the severe hypoxemia group than in the other two groups (p < 0.001).

**Table 3:** Characteristics of patients stratified by p/F during hospitalization

	N available observations	p/F ≥ 300 (N = 28)	p/F < 300 ≥ 200 (n = 27)	p/F < 200 (n = 21)	*p* value
Age (years)	76	52.3 (14.0)	59.2 (12.2)	63.1 (11.9)	0.014
Sex	76				0.618
Male		19 (68)	17 (63)	16 (76)	
Female		9 (32)	10 (37)	5 (24)	
BMI (Kg/m^2^)	75	25.7 (5.1)	27.3 (3.9)	28.0 (4.3)	0.181
p/F worst	76	349.0 (55.8)*	276.0 (54.0)*	135.0 (92.5)*	< 0.001
Hospitalization time (days)	76	9.5 (6.0)*	13.0 (9.0)*	23.0 (14.0)*	< 0.001
ICU admission	76	0 (0)	1 (4)	13 (62)	< 0.001
Smoking history	75				0.595
Never smoker		10 (36)	9 (33)	10 (50)	
Smoker		1 (3)	2 (8)	0 (0)	
Former smoker		17 (61)	16 (59)	10 (50)	
Pulmonary disease history					
COPD	76	0 (0)	2 (7)	0 (0)	0.155
Asthma	76	6 (21)	1 (4)	0 (0)	0.017
Chest CT performed	76	15 (54)	17 (63)	13 (62)	0.745
Radiology (chest XR or CT)	76				0.001
Unilateral involvement		11 (39)	3 (11)	0 (0)	
Bilateral involvement		17 (61)	24 (89)	21 (100)	
Antiviral therapy					
Lopinavir/Ritonavir	75	12 (43)	9 (35)	13 (62)	0.165
Darunavir/Ritonavir	76	14 (50)	19 (70)	13 (62)	0.300
Anti IL-6	76	2 (7)	10 (37)	17 (81)	< 0.001
Enoxaparin	76	8 (29)	11 (41)	20 (95)	< 0.001
Azithromycin	75	12 (43)	13 (50)	12 (57)	0.611
Hydroxychloroquine	76	27 (96)	25 (93)	21 (100)	0.422
Corticosteroids	74	0 (0)	3 (11)	2 (11)	0.195
Respiratory support					
Ventimask	76	8 (28)	25 (93)	21 (100)	< 0.001
HFNC	75	0 (0)	1 (4)	8 (38)	< 0.001
NIV or CPAP	75	0 (0)	0 (0)	11 (52)	< 0.001
Orotracheal Intubation	68	0 (0)	0 (0)	5 (25)	0.002
FVC^§^	73				
Litres		4.23 (0.18)	3.77 (0.18)	3.68 (0.21)	0.099
% predicted		119.6 (3.3)	104.5 (3.4)	92.0 (3.9)°	0.005
FEV_1_^§^	73				
Litres		3.36 (0.14)	3.00 (0.14)	2.98 (0.16)	0.110
% predicted		107.8 (3.0)	103.0 (3.1)	92.6 (3.6)°	0.009
FEV_1_/FVC^§^	73				
%		80.0 (1.0)	79.3 (1.0)	81.1 (1.2)	0.536
TLC^§^	72				
Litres		5.95 (0.23)	5.53 (0.24)	5.31 (0.26)	0.191
% predicted		92.6 (2.7)	90.7 (2.8)	80.4 (3.1)° ^#^	0.012
DL_CO_^§^	73				
Litres		23.23 (0.97)	21.05 (1.00)	18.69 (1.15)°	0.017
% predicted		82.7 (2.7)	80.6 (2.8)	64.9 (3.2)° ^#^	< 0.001
RV^§^	72				
Litres		1.58 (0.48)*	1.58 (0.44)*	1.48 (0.71)*	0.362
% predicted		77.6 (3.6)	73.8 (3.8)	70.8 (4.2)	0.498
ABG^§^	74				
pO_2_ (mmHg)		93.3 (1.6)	92.7 (1.5)	87.8 (1.8)	0.053
pCO_2_ (mmHg)		39.2 (0.6)	38.3 (0.6)	38.9 (0.7)	0.467
pH		7.41 (0.03)*	7.41 (0.04)*	7.42 (0.03)*	0.995
d(A-a) (mmHg)		10.1 (1.4)	12.3 (1.3)	16.6 (1.6)°	0.011
6MWT^§^	72				
SpO_2_ basal		98.0 (1.0)*	97.0 (2.0)*	97.0 (2.0)*	0.121
SpO_2_ nadir		96.5 (3.0)*	95.0 (3.0)*	94.0 (4.0)*°	0.005
Meters		560 (130)*	500 (95)*°	480 (140)*°	0.004
Dyspnoea VAS (mm)^§^					
Before	75	3.5 (2.1)	6.1 (2.1)	1.5 (2.5)	0.359
During	75	44.1 (5.4)	45.1 (5.5)	60.0 (6.4)	0.128
Follow-up	75	14.1 (3.5)	22.5 (3.6)	25.9 (4.1)	0.077
Cough VAS (mm)^§^					
Before	75	0.4 (1.3)	4.8 (1.4)	1.1 (1.6)	0.055
During	75	37.0 (5.8)	35.1 (5.9)	31.5 (6.9)	0.830
Follow-up	75	2.9 (2.3)	8.8 (2.3)	7.7 (2.7)	0.171

Data are presented as counts (%) or means (SD) or medians with interquartile range (IQR) for non-normally distributed variables (indicated with *). BMI: Body Mass Index; ICU: Intensive Care Unit; COPD: Chronic Obstructive Pulmonary Disease; IL-6: Interleukin; HFNC: High Flow Nasal Cannula; NIV: Non-Invasive Ventilation; CPAP: Continue Positive Airway Pressure; VAS: Visual Analogic Scale. ^§^Pulmonary Function, ABG, 6MWT parameters, Dyspnoea Visual Anlogic Scale (VAS) and Cough VAS are reported as estimated means (Standard Error) after adjustment for age used as covariate in the ANOVA model. °p value < 0.05 versus p/F ≥ 300 group. ^#^ p value < 0.05 versus p/F < 300 ≥ 200 group

As expected, the alveolar-arterial oxygen gradient increased progressively across study groups, ranging from 10.1 mmHg (± 1.4) in the mild hypoxemia group to 16.6 mmHg (± 1.6) in the severe hypoxemia group (p = 0.011). Compared to patients in the severe hypoxemia group, patients in the mild hypoxemia group demonstrated greater exercise tolerance (+ 80.0 m in 6MWD; p = 0.004) and higher nadir in SpO2 (+ 2.5%; p = 0.005). Dyspnoea and cough levels at the time of study visit were similar across groups.

## Discussion

The findings of this study suggest that respiratory abnormalities persist over time in COVID-19 patients who experienced a more severe form of disease during hospitalization. Several studies already reported a reduction in lung volumes and DLco levels as well as reduced exercise tolerance following hospital discharge [4–7]. Our study expands these findings in a larger Italian cohort. To our knowledge, this is the first study establishing the relationships between the severity of acute respiratory failure (as measured by the p/F ratio) and a wide range of blood gas and physiological parameters.

We identified a persistence of dyspnoea in the overall study population, a finding consistent with a study by Wong and coworkers, which reported dyspnoea in half of 78 COVID-19 patients after hospital discharge [13].

In order to explore the impact of disease severity on residual respiratory abnormalities, patients were stratified into three groups, according to levels of respiratory failure during hospitalization. No significant differences were observed regarding therapies, except for enoxaparin and anti-IL-6 drugs, administered more frequently in the severe group. The limited use of corticosteroids was likely due to the fact that evidence for dexamethasone use appeared towards the end of study completion [14]. We cannot exclude that a more extensive use of corticosteroids would have changed our findings.

Patients with mild and moderate disease had normal lung volumes. In contrast, a mild reduction in RV was found in the severe hypoxemia group. Whether this finding results by altered lung compliance in this group [15] remain to be determined. Moreover, TLC was at the lower limit of normal in the severe group: this finding suggests a link between severity of COVID-19 pneumonia and reduction in lung volumes. Whether such abnormalities were due to the presence of fibrotic sequelae after acute interstitial pneumonia could not be determined, since our cohort did not undergo a chest CT scan at the time of the study visit. Moreover, we identified normal DLco values in the mild and the moderate hypoxemia groups and reduced values in the severe hypoxemia group. This could reflect the degree of microvascular and epithelial damage, likely to be more consistent in the severe cases [16]. Patients recovering from ARDS from any cause may have persistent functional impairment one year after hospital discharge [17]. Therefore, these findings might not be COVID-19-specific.

Our study had several limitations. CT imaging was not available at the time of study visit: as such, the relationships between functional impairment and residual fibrotic changes remain unknown. The follow-up time in this study is short, and further studies are warranted to clarify whether respiratory abnormalities persist in the longer term. The use of p/F ratio to classify COVID-19 severity is not ideal as it may not be reliable in non-intubated patients [18]. Finally, the levels of dyspnoea and cough before and during hospitalization were collected at the time of the follow-up clinical evaluation: they may therefore not measure accurately the severity of symptoms at those timepoints.

## Conclusion

Severe COVID-19 pneumonia may result in respiratory abnormalities and a reduction in exercise tolerance, which can be present at least one month after hospital discharge. Moreover, a low p/F ratio during the acute phase of the infection seems to correlate with a residual reduction of lung volumes, and residual reduction in DLCO. Further follow up is required to determine the degree of pulmonary and exercise impairment following hospitalization for COVID-19 pneumonia.

## Data Availability

The datasets used and/or analysed during the current study are available from the corresponding author on reasonable request.

## Abbreviations

SARS: Severe acute respiratory syndrome
CRP: C-reactive protein
ARDS: Acute distress respiratory syndrome
ICU: Intensive Care Unit
6MWD: Six-minute walk distance
6MWT: Six-minute walk test
p/F: PaO_2_/FiO_2_ ratio
ABG: Arterial blood gas analysis
PFT: Pulmonary function tests
DLco: Diffusing capacity of the lung for carbon monoxide
SpO_2_: Percutaneous oxygen saturation
HR: Heart rate
VAS: Visual Analog Scale
COPD: Chronic Obstructive Pulmonary Disease
NIV: Non-Invasive Ventilation
CPAP: Continuous Positive Airway Pressure
TLC: Total lung capacity
FVC: Forced vital capacity
FEV-1: Forced expiratory in the first second
RV: Residual volume
pO_2_: Partial pressure of oxygen
d(A-a): Alveolar-arteriosus oxygen gradient
